# MUC4-ErbB2 Oncogenic Complex: Binding studies using Microscale Thermophoresis

**DOI:** 10.1038/s41598-019-53099-0

**Published:** 2019-11-13

**Authors:** Maxime Liberelle, Romain Magnez, Xavier Thuru, Yamina Bencheikh, Severine Ravez, Camille Quenon, Anne-Sophie Drucbert, Catherine Foulon, Patricia Melnyk, Isabelle Van Seuningen, Nicolas Lebègue

**Affiliations:** 1 Univ. Lille, Inserm, CHU Lille, UMR-S1172 – JPArc – Centre de Recherche Jean-Pierre Aubert Neurosciences et Cancer, F-59000 Lille, France; 2 CHU Lille, Banque de tissus, F-59000 Lille, France; 3 Univ. Lille, EA 7365, GRITA - Groupe de Recherche sur les formes Injectables et les Technologies Associées, F-59000 Lille, France

**Keywords:** Thermodynamics, Pancreatic cancer

## Abstract

The MUC4 membrane-bound mucin is a large O-glycoprotein involved in epithelial homeostasis. At the cancer cell surface MUC4 interacts with ErbB2 receptor via EGF domains to promote cell proliferation and migration. MUC4 is highly regarded as a therapeutic target in pancreatic cancer as it is not expressed in healthy pancreas, while it is neoexpressed in early preneoplastic stages (PanINs). However, the association/dissociation constant of MUC4-ErbB2 complex is unknown. Protein-protein interactions (PPIs) have become a major area of research in the past years and the characterization of their interactions, especially by biophysical methods, is intensively used in drug discovery. To characterize the MUC4-ErbB2 interaction, we used MicroScale Thermophoresis (MST), a powerful method for quantitative protein interaction analysis under challenging conditions. We worked with CHO cell lysates containing either the transmembrane β subunit of MUC4 (MUC4β) or a truncated mutant encompassing only the EGF domains (MUC4_EGF3+1+2_). MST studies have led to the characterization of equilibrium dissociation constants (K_d_) for MUC4β-ErbB2 (7–25 nM) and MUC4_EGF3+1+2_/ErbB2 (65–79 nM) complexes. This work provides new information regarding the MUC4-ErbB2 interaction at the biophysical level and also confirms that the presence of the three EGF domains of MUC4 is sufficient to provide efficient interaction. This technological approach will be very useful in the future to validate small molecule binding affinities targeting MUC4-ErbB2 complex for drug discovery development in cancer. It will also be of high interest for the other known membrane mucins forming oncogenic complexes with ErbBs at the cancer cell surface.

## Introduction

## Context

**Figure 1:**
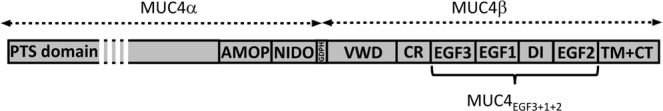
Schematic view of MUC4. Schematic representation of the membrane-bound mucin MUC4 featuring both subunits MUC4α and MUC4β. MUC4α is the mucin-like subunit, with a 27 amino-acid (aa) long signal peptide, an approximate 126–130 aa repeat domain, a 554 aa domain and a 16 aa sequence, variably repeated from 145 to 345 times and rich in proline/serine/threonine residues (PST domain), strongly O-glycosylated. Two protein domains are then found, the AMOP and the NIDO domains. The subunits are post-transcriptionally cleaved by an auto-cleavable GDPH sequence and non-covalently associated. MUC4β is formed by a von Willebrand factor-D (VWD) followed by an uncharacterized part, strongly N-glycosylated and by a cysteine-rich domain (CR). Two EGF-like domains come next, EGF3 and EGF1 separated by a short linker. Then an intermediate uncharacterized domain (DI), another EGF-like domain EGF2 and a transmembrane helix (TM) with a short cytoplasmic tail (CT).

Mucins form a family of large O-glycoproteins, of heterogeneous evolutionary origin, organized into a peptidic chain called apomucin in which a Serine/Threonine-rich region is intensively glycosylated. In addition to this O-glycosylation, which represents up to 50–80% of the total molecular weight of the protein, N-glycosylation may be present to a lesser extent^1^. MUC4 belongs to the membrane-bound mucin class and is synthesized as a single chain that may be cleaved into two subunits tightly associated by non-covalent interactions. MUC4α is the extracellular mucin-like O-glycosylated subunit whereas MUC4β is the membrane-tethered subunit. MUC4β contains several functional domains, such as epidermal growth factor (EGF)-like domains, Von Willebrand factor type D (VWD) domain and a single transmembrane helix (TM) with a short cytoplasmic tail (CT)^2^ (Fig. 1). Membrane-bound mucins have been reported to be involved in pathological disorders and particularly in neoplastic development of cancers^3,4^.

MUC4 is indeed extensively regarded as an overexpressed pro-tumorigenic protein in epithelial cancers (such as lung, esophagus, colon, breast or pancreas^5^) as it forms an oncogenic complex with ErbB2 receptor tyrosine kinase. The physical interaction between human MUC4 and ErbB2 involves a region of the extracellular domain of MUC4β composed of three EGF-like domains^6^. Two of the three EGF-like domains (EGF1 and EGF2), conserved throughout evolution, are structurally equivalent to human EGF and biologically active^7–10^. Moreover, previous studies with rat ortholog of MUC4 sialomucin complex have shown the beneficial effect of EGF1-like domain in the direct interaction with ErbB2^7^.

Among the ErbB family, ErbB2 appears as a keystone for ErbB signaling, as it forms the most potent complex. It is the favored dimerization forming partner^11,12^, and is a key feature for crosstalk between numerous cells signaling pathways^13,14^. More than two decades of studies have shown that ErbB2 is one of the most common overexpressed oncogenic factors with a strong correlation between its amplification and the aggressiveness of the tumor^15–18^. Numerous therapeutic approaches are targeting ErbB2, either based on antibodies targeting epitopes or on inhibitors targeting the catalytic domain. Unfortunately, these treatments are expensive, drive resistances, are associated with several side effects^19,20^ and in pancreatic cancer they often fail. Next to that, *in vitro* and *in vivo* data in pancreatic cancer show that silencing of MUC4 expression results in altered tumor cell behavior, decreased growth, decreased ErbB2 expression and a marked reduction in metastatic incidence^6,21,22^. In that context, targeting MUC4 thus appears as a new promising therapeutic approach for the development of small inhibitor to treat epithelial cancers associated with MUC4-ErbB2 overexpression^10,22,23^, when ErbB2 targeting has failed.

In this study, we aimed at developing *in vitro* biophysical assays for macromolecular binding characterization between MUC4β or MUC4_EGF3+1+2_ (minimal MUC4 sequence for interaction with ErbB2 as shown previously, Fig. 1)^6^ and ErbB2. In addition to providing an equilibrium dissociation constant (K_d_) value for the first time, our work led to methodologies allowing characterization at the molecular level of this complex *in vitro*. We also aimed at developing a challenging purification-free strategy for low-protein concentration samples (such as total cell lysates), to avoid time and resource consuming steps which are often labor consuming using MicroScale Thermophoresis (MST)^24–26^ technological approach.

MST is a powerful biophysical method which has already proved useful and efficient in our laboratory to characterize binding affinities of ligand-protein^27^ or protein-protein^28^ interactions as therapeutic targets. In this study, we provided, a highly focused method combining fluorescent fusion protein, cell lysate and recombinant ErbB2 by using MST.

## Results

### MST studies

#### Buffer optimization

**Figure 2:**
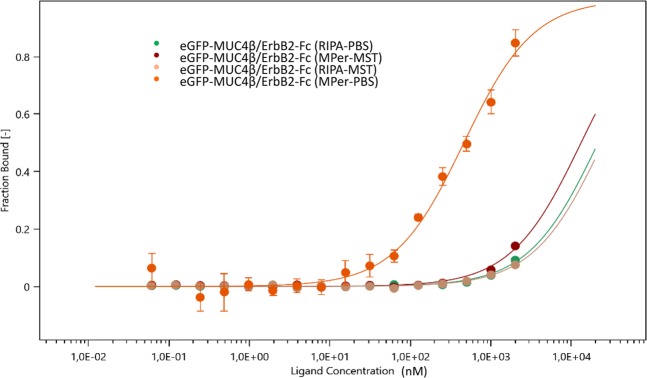
Effect of different combinations of lysis/dilution buffer on the binding curve of MUC4-ErbB2 in MST. Combinations of lysis/dilution buffer are given in this order. Binding curves of eGFP-MUC4β with ErbB2-Fc at 23 °C in RIPA-MST (pink curve), in RIPA-PBS (green curve) or MPer-MST (dark red curve) lead to K_d_ > 20 µM with no saturation. The binding curve in MPer-PBS condition (orange) allowed to find a K_d_ value in the range of several hundred nanomolars.

To develop the MST strategy, fluorescent eGFP tag fused to MUC4β was expressed in CHO-K1 cells and used without further purification. Commercially available recombinant ErbB2, used as the binding partner, remained label-free. CHO-K1 cell extracts expressing eGFP-MUC4β fusion proteins were first obtained using RIPA buffer. Cell extracts were then diluted either with PBS or a specific Tris-based MST buffer provided by NanoTemper^®^ technologies. This led us to reach an optimum fluorescence level to approximatively 500 FI units, with final concentrations between 30 and 50 nM that were calculated from a fluorescence calibration curve^28^. As no significant interaction with recombinant ErbB2 was measured under these conditions (Fig. 2, green and pink curves), lysis buffers were changed to less stringent buffers as described in NanoTemper^®^ guidelines. Cell extraction and dilutions were then performed in optimized conditions, respectively with MPer and PBS buffers and led to a fitted binding curve with tolerance of 20% from unvarying fluorescence among the sixteen capillaries (Fig. 2, orange curve). These results indicated that buffers containing Tris (RIPA, MST) were not suited for such interactions studies whereas those containing bicine (MPer) combined with PBS were well-suited.

#### MST assay optimization

MST measurement conditions were next studied to find the optimum IR laser heating time and power. No sign of aggregation, adhesion or convection phenomenon were observed with laser off/on times of 30 s and 80% MST power. As a very well defined equilibrium state could be reached, the value for the K_d_ remained stable until the laser went off at 30 s (Fig. S1). Impact of MST power was also studied to provide K_d_ values with great fitted binding curves, weak background signals and amplitude signals 3-fold greater than the noise level (Fig. S2). 80% MST power gave the best response, in accordance with the literature describing MST experiments on PPIs in biological fluids^29^.

#### Incubation time optimization

Since MUC4 is produced as a soluble protein in our conditions, this may lead to misfolded protein that needs pre-incubation time to better interact with ErbB2. Binding profile evolution was thus monitored after protein incubation (MUC4β or MUC4_EGF3+1+2_ and ErbB2) at room temperature and MST experiments carried out every half hour (Fig. S3). For both MUC4 proteins, incubation time was found optimal from a period ranging between three and five hours at room temperature or 24 h at 4 °C. This was followed by a loss of affinity as a result of lysate degradation. Thus, MUC4β and MUC4_EGF3+1+2_ behaved similarly.

#### Measurement of MUC4β-ErbB2-Fc interaction

**Figure 3:**
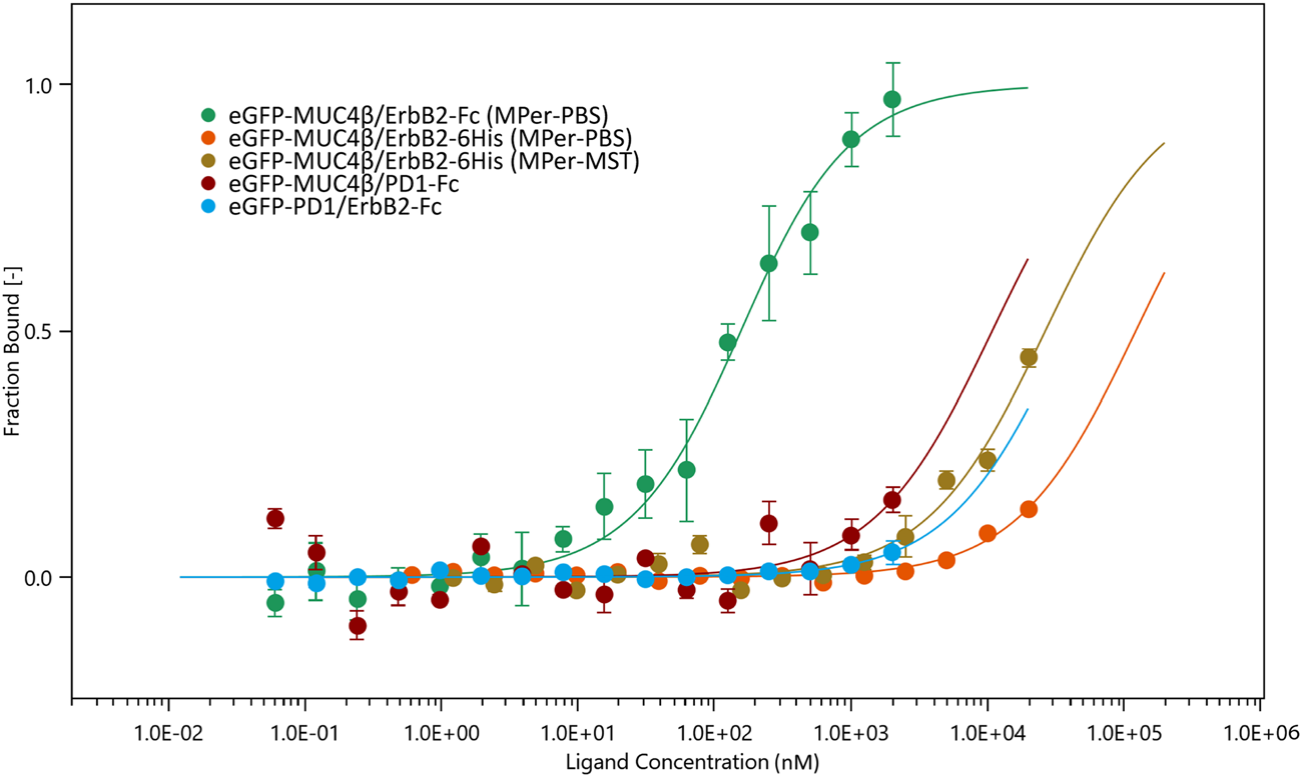
Binding curves and specificity controls. No significant interaction (K_d_ > 20 µM) was found for MUC4β with a non-relevant protein containing the same Fc (dark red curve) than recombinant ErbB2 nor for ErbB2 with a non-relevant eGFP fusion protein (blue curve), in comparison with the specific interaction between MUC4β and ErbB2 (green curve). This curve displays the exact dose-response curve for the binding interaction between ErbB2-Fc and eGFP-MUC4β lysate, with the concentration of eGFP-MUC4β proteins kept constant around 33 nM while the ErbB2-Fc concentration varies from 1 μM and 0.05 nM. This binding curve yields to a K_d_ of 131 ± 24 nM on two distinct cell lysates and performed each in triplicate. In comparison the dose-response curves in two different buffers (orange and brown curves) for the binding interaction between ErbB2-6His and eGFP-MUC4β lysate. The concentrations of ErbB2-6His range from 20 µM to 0.61 nM.

Two negative cross-controls were performed to confirm the absence of non-specific interactions with non-relevant proteins. No binding curves occurred between recombinant ErbB2 and non-relevant eGFP fusion protein (eGFP-PD-1) nor between eGFP-MUC4β and ErbB2-Fc-like domain-containing protein (PD-1-Fc) (Fig. 3). The optimized experimental conditions allowed the measurement of the binding affinity for MUC4β-ErbB2-Fc with a K_d_ value of 131 ± 24 nM and a total amplitude of 60 (Fig. 3, green curve). Applying the same conditions with recombinant ErbB2 (ErbB2-6His, mainly monomeric) and MUC4β did not lead to any measurable interaction either in PBS or in MST buffer (Fig. 3).

**Figure 4:**
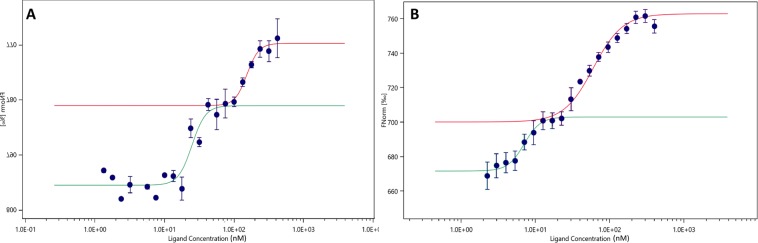
Improved binding curves for interaction of eGFP-MUC4β and ErbB2-Fc in normal and reverse mode. (**A**) Dose-response curve in triplicate for the binding interaction between ErbB2-Fc and eGFP-MUC4β lysate, with a dilution ratio of 3:1 instead of 1:1, all other parameters being kept optimal. The curve obtained presents two distinguishable binding events that can be approximated with a Hill model fitting. The first (green curve) is around 25 ± 5 nM and the second (red curve) spawn at 152 ± 80 nM. (**B**) Dose-response curve in triplicate for the binding interaction between ErbB2-Fc tagged by red fluorescent anti-HIS dye and eGFP-MUC4β lysate, with a dilution ration of 3:1. MUC4 concentrations range from 2.27 nM to 403 nM. The curve obtained presents two distinguishable binding events that can be approximated with a Hill model fitting. The first (green curve) is around 7 ± 5 nM and the second (red curve) spawns at 55 ± 17 nM.

To then improve the fitted binding curve quality, a tighter range of dilutions, allowing 32 point measurements in the same concentration range, was performed. The resulting dose-response curve showed two distinguishable binding events that could be estimated with a Hill model fitting. The first binding event provided a K_d_ close to 25 ± 5 nM and the second event, with a lower affinity, a K_d_ at 152 ± 80 nM (Fig. 4A). To confirm these results, and the occurrence of the two binding events, a reverse model was designed with ErbB2 labelled with a fluorophore emitting in the red wavelength and at a constant concentration. Cell lysates containing eGFP-MUC4β were then used as ligands. This methodology using cell lysates as ligands is original and innovative and has not yet been described to date. As for the normal model (Fig. 4A), a dual profile was obtained with two binding events displaying slightly better affinities in the range of 7 ± 5 and 55 ± 17 nM, respectively (Fig. 4B).

#### Involvement of MUC4β EGF domains in the interaction

**Figure 5:**
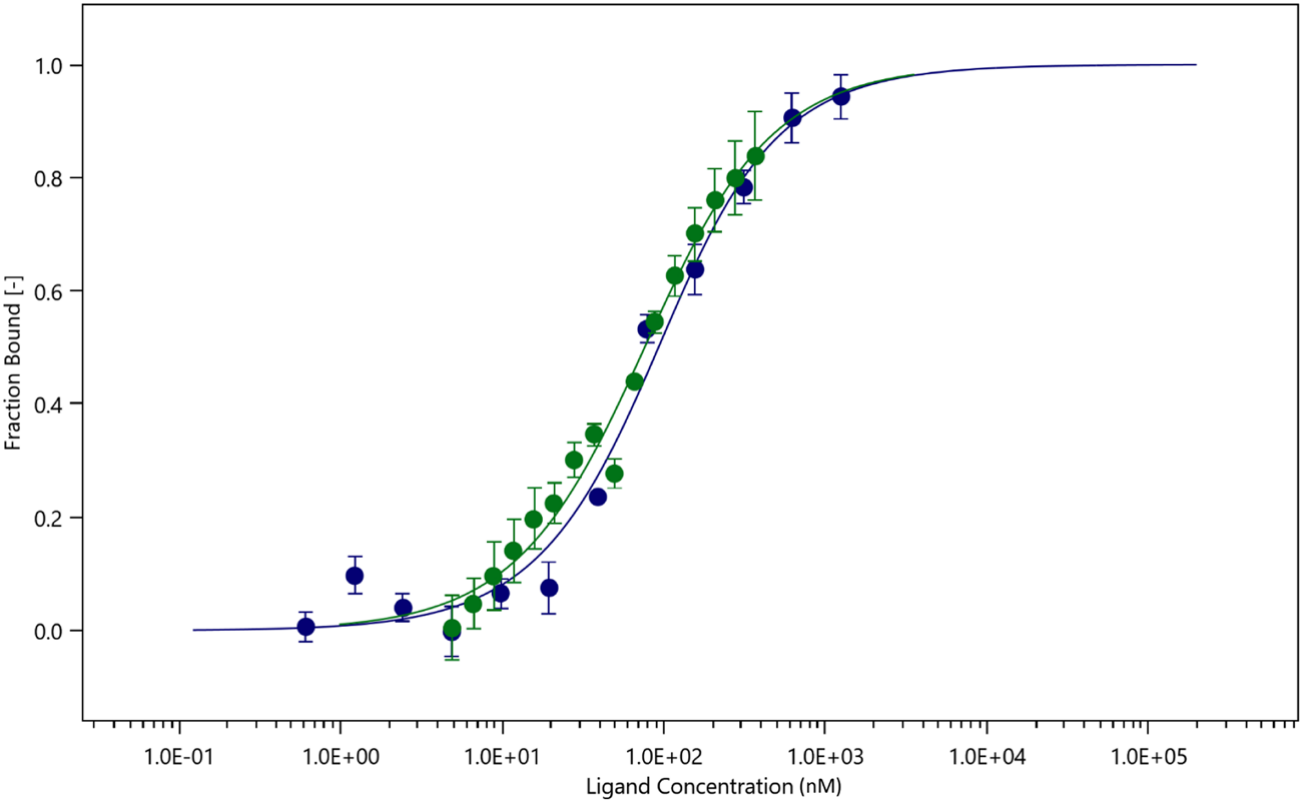
Dose-response binding curves in normal and reverse mode for interaction of eGFP-MUC4_EGF3+1+2_ and ErbB2-Fc. The blue curve corresponds to the normal mode, following the fluorescence of the eGFP tagged protein with titration of ErbB2-Fc. The green curve corresponds to the reverse mode with ErbB2 tagged to be red fluorescent and titrated against eGFP-MUC4_EGF3+1+2_. While the concentration of eGFP-MUC4_EGF3+1+2_ protein is kept constant at 33 nM, the ErbB2-Fc concentration ranged from 1 μM and 0.05 nM. While the concentration of ErbB2-Fc is kept constant at 50 nM, the eGFP-MUC4_EGF3+1+2_ concentration ranged from 4,9 nM to 370 nM. The normal mode binding curve yields a K_d_ of 79 ± 18 nM while the reverse mode yields a K_d_ of 65 ± 14 nM.

We showed previously that the physical interaction between MUC4β and ErbB2 had involved a region encompassing the three EGF-like domains present in MUC4β^6^. To confirm this result, CHO-K1 cell lysates expressing eGFP-MUC4_EGF3+1+2_ were then used either with ErbB2 as a ligand or in the original reverse model. These experiments led to two binding curves, with only one binding event, which almost overlapped each other, providing K_d_ values of 79 ± 18 nM and 65 ± 14 nM, for the normal and reverse models respectively (Fig. 5).

## Discussion

Membrane-bound mucins, which are overexpressed in numerous epithelial cancers, have been considered as oncogenic factors for many years but the molecular mechanisms by which they mediate their oncogenic activities at the membrane are still unknown^3,5,22,30–33^. MUC4 and more recently MUC3 and MUC13 have been described as membrane partners for the well-known orphan receptor ErbB2^22,34,35^, which is often overexpressed in numerous cancers^36–40^. ErbB2 targeted therapies, using antibodies or catalytic inhibitors, remain poorly efficient in pancreatic cancer, and have also been associated with ill side effects in other cancers^41–43^. In this context, the inhibition of the oncogenic activity by developing MUC4β-ErbB2 protein-protein interaction (PPI) modulators appears as a promising therapeutic alternative in pancreatic cancer. This approach may also be applied to other epithelial cancers where overexpression of the MUC4-ErbB2 complex is observed (lung, esophagus, stomach, colon…) and ErbB2 targeting has failed. The major challenges in developing PPI modulators consist in structural characterization of the complex and development of a direct evaluation process. Recently, human MUC4-ErbB2 complex has started to be understood at the molecular level by biochemical studies^6^ but no structural data or PPI quantification have been available up to now. Development of biophysical assays allowing MUC4-ErbB2 PPI quantification and understanding the structure-function relationship of the complex is thus of strategic importance regarding the functionality of ErbB2 receptor. This may lead to the discovery of new anti-cancer therapeutic drugs.

We chose to develop an MST assay since this technology, routinely used in our team^27,28^, does not require immobilization, requires low sample amounts, and can be used for biological fluids. The only drawback is that it needs fluorescent labelling which may cause non-specific binding. This approach showed that MST is highly protein-dependent in terms of lysis and dilution buffer. Tris is the most common buffer used in biochemical and biophysical studies but appears to behave negatively for proper protein folding^44^ and promotes aggregation^45^. Combination of bicine lysis buffer and phosphate dilution buffer is the most efficient for lowering those detrimental effects and this was confirmed in the setting up of our experiments. MUC4β recombinant protein was designed with its transmembrane α helix and was produced as a cytoplasmic protein not anchored to the cell membrane. This type of production may lead to misfolded protein which then needs long incubation times to interact with recombinant human ErbB2-Fc chimera. Furthermore, this ErbB2-Fc is described as a disulfide-linked homodimeric protein expressing the extracellular domains that lacks the TM and CT part. These two characteristics may explain the long incubation times required to produce a perfectly folded complex but which could also be linked to the intrinsic property of the interaction between ErbB2 and MUC4.

This work provides the first interaction affinity measurement involving the oncogenic ErbB2 protein tyrosine kinase receptor and its membrane partner the MUC4 mucin. It also provides an optimized methodology suitable for studying ErbB2 with other potential membrane partners. In all MST experiments, two binding events were observed between MUC4β and ErbB2. The first event displayed high affinities ranging from 7 to 25 ± 5 nM and the second interaction phase providing lower affinities ranging from 55 ± 17 to 152 ± 80 nM. Interestingly, the binding curves between MUC4_EGF3+1+2_ and ErbB2 displayed only one interaction phase with K_d_ values ranging from 65 to 79 ± 18 nM. These results suggest that the second event of low affinity may represent an interaction between the non-EGF-like domains of MUC4β and ErbB2. This may involve the C-terminal transmembrane helix (TM) and its short cytoplasmic tail (CT) or the VWD domain at the N-terminal part of MUC4β (see Fig. 1). These results also confirm that the physical interaction between MUC4β and ErbB2 is mediated for the major part by the EGF-like domains of MUC4β and provides the first mechanistic insights about the MUC4-ErbB2 structure-function relationship^6^. The presence of dual binding events will need further studies to determine whether other determinants are involved in the interaction and which role are played by the non-EGF-like domains.

Finally, the importance of the dimeric character of ErbB2 was studied as the ErbB family members are known to have a tight relationship between the dimerization and the binding of ligands. For this purpose, MST experiments involving MUC4β and recombinant monomeric ErbB2 (ErbB2-6His) were performed but did not lead to any binding events. This result rigorously confirmed, for the first time, that the MUC4-ErbB2 interaction displays similar behavior as the rest of the ErbB receptor family complexes which require dimeric forms to be active^46–48^. This methodological approach could thus be used to study the interaction of the other membrane-bound mucins involved in cancer progression that are known to interact with ErbB receptors^10^.

SPR, one of the main biophysical method broadly used in drug discovery, was tested as an orthogonal method to MST with the additional benefit of providing access to the kinetic parameters of the interaction. Unfortunately, SPR needed high ErbB2 sample consumption and did not appear to be adapted for studying the specific characteristics of the MUC4β-ErbB2 which needs long incubation times not appropriate for a microfluidic system (see supplementary SPR studies).

In conclusion, we have developed a convenient and efficient method allowing MUC4-ErbB2 PPI quantification by microscale thermophoresis. This purification free strategy from cell lysates overexpressing eGFP fusion proteins led to binding affinities regardless of which partner is labelled with a fluorescent tag. Future studies will be now directed on the structure-function relationship of MUC4-ErbB2 complex allowing critical insights for EGF domain targeting, to better inhibit the complex formation. Recent studies have shown that MST is suited for compound screening with a better output than other methods^49,50^ and thus comfort us with this strategy. Having validated this method to measure K_d_ values, we will now continue our work aiming at developing small inhibitory therapeutic molecules targeting MUC4 by the means of MST fragment or chemical library screening to identify ligands. This should open new avenues regarding MUC4 potential as a therapeutic target in cancer.

## Methods

### Materials

Reagents were obtained as follows: human recombinant ErbB2 protein (5 mg, R&D systems, ref. 1129-ER), Amaxa® Cell Line Nucleofector® Kit T (Lonza), RIPA lysis buffer, MPer lysis buffer and Halt^TM^ Protease Inhibitor Cocktail (Thermo Scientific™), MST buffer, Tween 20, Monolith His-Tag Labeling Kit RED-tris-NTA 2nd Generation and premium coated capillaries (Nanotemper™), recombinant MUC4β and MUC4_EGF3+1+2_ inside peGFP-C1 vector (ProteoGenix SAS Schiltigheim, France).

### eGFP-MUC4 constructs

The sequence for the MUC4 (accession number #Q99102) protein was obtained from Uniprot. Only the β subunit was kept, without the four amino acids involved in cleavage (GDPH), in order to obtain a monomeric eGFP-MUC4β fusion protein (optimized for CHO-K1 expression and synthesized by ProteoGenix SAS). DNA sequence was cloned into the peGFP-C1 vector using the appropriate restriction sites, with kanamycin and neomycin resistance genes (Fig. S7). MUC4_EGF3+1+2_ construct was designed and produced by ProteoGenix SAS with the same specifications, considering the domain limits from Uniprot (from the start of EGF1 domain to the end of EGF2 domain), based on data from our previous work^6^.

### Plasmid amplification and purification

Mix & Go competent E. coli cells (Zymo Research) were used for the cloning of DNA fragments and the preparation of plasmids because they possess recA1 and endA1 gene mutations that increase the insert stability and the extracted DNA quality. The strains were stored at −80 °C. The peGFP-C1 Kana/MUC4β and the peGFP-C1 Kana/MUC4_EGF3+1+2_ plasmids were transformed into E. coli. The cultures were scaled-up for Midi-prep and DNA was extracted with the Plasmid DNA purification kit (Nucleobond Xtra Midi/Maxi kit). The plasmid concentration was quantified using NanoDrop® spectrophotometer (ThermoFischer Scientific). Optimized DNA concentration (2 μg.μL^−1^) was used for cell transfection studies^28^.

### Cell culture

Transfected CHO-K1 cells were grown in Ham’s F12 medium supplemented with 10% (v/v) heat-inactivated fetal bovine serum (Life Technologies) and 1% (v/v) Penicillin-Streptomycin (Life Technologies). Cells were incubated at 37 °C in a humidified atmosphere with 5% CO_2_ and maintained using standard cell culture techniques.

### Cell transfection

peGFP-C1 plasmids encoding MUC4β or MUC4EGF_3+1+2_ were transfected into CHO-K1 cells (ATCC® CCL-61™) using the Cell Line Nucleofector® Kit T. One million cells were used with 2 µg of plasmid at 2 µg.µL^−1^ in 100 µl of the Nucleofector solution. After 48 h, appropriate selective pressure was applied to the culture medium for 14 days to select transfected cells. These latter were then separated as previously described by flow cytometry and kept for thermophoresis analyses^28^.

### Cell lysis

CHO-K1 peGFP-C1 transfected cells were treated at confluence. After washing with PBS and trypsinization, cells were centrifuged at 1400 rpm for 5 min. After removing the supernatant, cells were washed with 5 ml PBS then centrifuged again for 5 min. Pelleted cells were then lysed using 300 µl of MPer-buffer containing 1% protease inhibitor cocktail (Halt™ Protease Inhibitor Cocktail, EDTA-free, Thermo Fisher Scientific) and incubated on ice for 30 min with vortexing every 15 min. The cell lysate was then processed three times in an ultrasonic bath for 30 s and after that centrifuged at 15000 rpm at 4 °C to remove large cell aggregates and cell debris. The supernatant (cell lysate) was recovered and stored at 4 °C for rapid and fresh use without freezing.

### GFP titration

To calculate the concentration of the eGFP fusion protein in the cell lysate, an in-house calibration curve was used as previously described^28^. The fluorescein calibration curve is carried out on the thermophoresis instrument by establishing a range with different concentration points (from 0 to 100 nM). Each concentration is linked to an amount of fluorescein fluorescence (FI Units). The concentration of the labeled protein eGFP is determined using relationship that connects the Fluorescein Units to the eGFP, their quantum yields, and the respective molar extinction coefficients at a given excitation length^28^. Since the FI Units (eGFP) were given by the initial fluorescence (cap-scan), it was possible to convert this fluorescence in its fluorescein equivalent. Reported on the calibration curve, this value allowed a determination of the concentration of the eGFP labeled protein.

### ErbB2 fluorescent tagging

100 nM of the ErbB2-Fc protein, also possessing a 6His-tag, was mixed with 50 nM of His-Tag Labeling Kit RED-tris-NTA (NanoTemper Technologies). After 30 min incubation, the protein solution was readily usable for thermophoresis measurement.

### MicroScale thermophoresis measurement

Dilution buffer was prepared with 1X PBS containing 0.05% (v/v) P20 (PBS-T). For the normal model, appropriate volume (5 to 10 µL) of ErbB2-Fc or PD-1-Fc (R&D systems) was diluted 1:1 in dilution buffer to make dilution series of titrated solutions. For the reverse model, 10 µL of high-concentrated lysates (containing eGFP-MUC4β, eGFP-MUC4_EGF3+1+2_) was diluted 3:1 to make dilution series of titrated solutions. For the titrant solutions, the cell lysates containing eGFP-fused proteins (eGFP-MUC4β, eGFP-MUC4_EGF3+1+2_, eGFP-PD-1) or the tagged ErbB2 with red-NTA dye were diluted in PBS-T buffer at a normalized fluorescence of 1000 FU at 100% LED power. Then equivalent volume of titrant and titrated solution were mixed to a final volume of 10 or 20 µl. The maximal concentration for ErbB2-Fc was 1 µM and the maximal concentration for containing eGFP-fused proteins was 400 nM. The initial fluorescence for each sample was 500 FU at 100% LED power, corresponding to a constant concentration in eGFP-protein of 35 nM and 25 nM for ErbB2. For measurements, samples were filled into premium coated capillaries (NanoTemper Technologies). The measurements were conducted on a NanoTemper Monolith NT.115 instrument (NanoTemper Technologies). The analysis was performed at 100% blue LED power for eGFP-fused; 100% red LED power for NTA dyed ErbB2 and all at 80% MST power, with a standard 5 *s* before, MST-on for 30 *s* and 5 *s* after MST-off.

## Supplementary information

Supplementary data
