# Publisher Correction: MUC4-ErbB2 Oncogenic Complex: Binding studies using Microscale Thermophoresis

**DOI:** 10.1038/s41598-020-63163-9

**Published:** 2020-04-14

**Authors:** Maxime Liberelle, Romain Magnez, Xavier Thuru, Yamina Bencheikh, Severine Ravez, Camille Quenon, Anne-Sophie Drucbert, Catherine Foulon, Patricia Melnyk, Isabelle Van Seuningen, Nicolas Lebègue

**Affiliations:** 1 Univ. Lille, Inserm, CHU Lille, UMR-S1172 – JPArc – Centre de Recherche Jean-Pierre Aubert Neurosciences et Cancer, F-59000 Lille, France; 2 CHU Lille, Banque de tissus, F-59000 Lille, France; 3 Univ. Lille, EA 7365, GRITA - Groupe de Recherche sur les formes Injectables et les Technologies Associées, F-59000 Lille, France

Correction to: *Scientific Reports* 10.1038/s41598-019-53099-0, published online 13 November 2019

In the original version of this Article, the author Isabelle Van Seuningen was incorrectly indexed. This error has now been corrected.

