# Supplementary material for: MUC4-ErbB2 Oncogenic Complex: Binding studies using Microscale Thermophoresis
Source: Sci Rep. 2019 Nov 13;9:16678. doi: 10.1038/s41598-019-53099-0 (PMC6853952; doi:10.1038/s41598-019-53099-0)
Supplement: Supplementary file 1 — Supplementary data [file 41598_2019_53099_MOESM1_ESM.pdf]

Supplementary Data

MUC4-ErbB2 Oncogenic Complex: Binding studies using Microscale Thermophoresis

Maxime Liberelle[1], Romain Magnez[1], Xavier Thuru[1], Yamina Bencheikh[1], Severine Ravez[1], Camille Quenon[1], Anne-Sophie Drucbert[2], Catherine Foulon[3], Patricia Melnyk[1], Isabelle Van Seuningen[1*], Nicolas Lebègue[1*]

[1] Univ. Lille, Inserm, CHU Lille, UMR-S1172 – JPArc – Centre de Recherche Jean-Pierre Aubert Neurosciences et Cancer, F-59000 Lille, France

[2]CHU Lille, Banque de tissus, F-59000, Lille France

[3]Univ. Lille, EA 7365, GRITA - Groupe de Recherche sur les formes Injectables et les Technologies Associées, F-59000, Lille, France.

Corresponding author: Pr Nicolas Lebègue, Inserm UMR-S 1172/JPARC, Faculté de Pharmacie, 3 rue du Pr. Laguesse, 59045 Lille, France, Phone: +33 3 20 96 49 77, email: nicolas.lebegue@univ-lille.fr

Supplementary Fig. S1

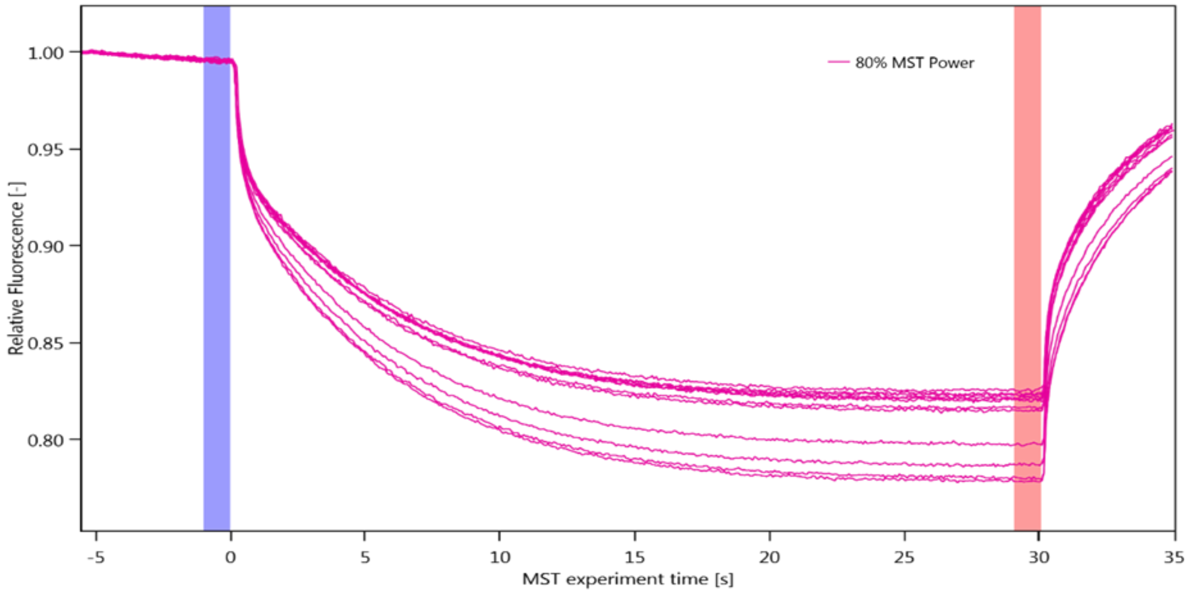

Thermograph analysis of MUC4β-ErbB2. Thermographs of eGFP-MUC4β in MPer-PBS buffer conditions binding to ErbB2 at 23 °C. After 30 sec of IR laser heating at 80 % MST power, no convection nor aggregation phenomenon occurred. This allowed proper determination of K_d in these unusually harsh conditions. Cold region is set to 0 *sec* (blue) and hot region to 30 *s* (orange).

Supplementary Fig. S2

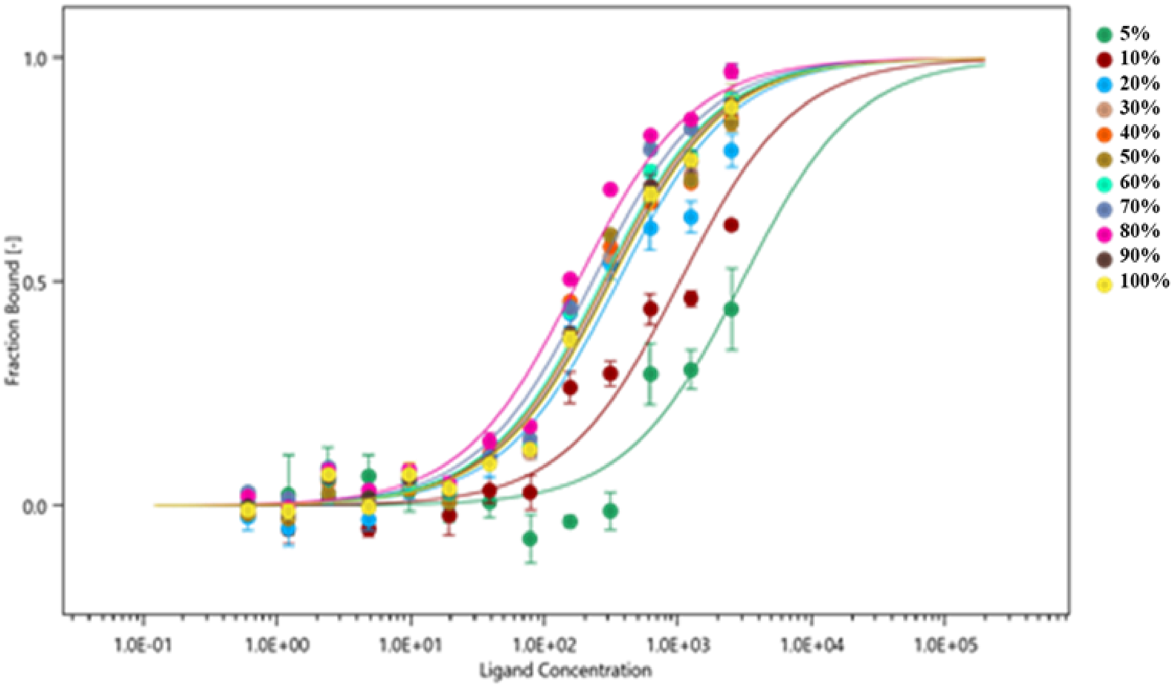

MST Power	Kd	Kd Confidence	Response Amplitude	n	Std. Error of Regression	Signal to Noise	Excitation Power
5.00%	1294,78	409,55	4,32	3	0,54	8,43	100%
10.00%	546,79	84,93	7,91	3	0,51	16,27	100%
20.00%	296,90	38,81	16,00	3	1,01	16,51	100%
30.00%	283,55	35,71	22,59	3	1,17	20,10	100%
40.00%	280,15	37,99	32,36	3	1,81	18,77	100%
50.00%	279,53	31,52	41,00	3	2,23	19,14	100%
60.00%	265,29	59,94	50,71	3	2,48	22,62	100%
70.00%	225,78	28,02	55,00	3	2,85	19,70	100%
80.00%	174,81	24,19	55,16	3	3,10	18,66	100%
90.00%	302,94	62,59	64,93	3	2,78	25,84	100%
100.00%	314,60	63,89	64,10	2	2,66	26,63	100%

Impact of the MST power on the MUC4β-ErbB2 K_d measurement. Comparison of various MST power toward the MUC4β-ErbB2 interaction. The best compromise is obtained at high irradiation power, with 80% having slightly better signal-to-noise ratio.

Supplementary Fig. S3

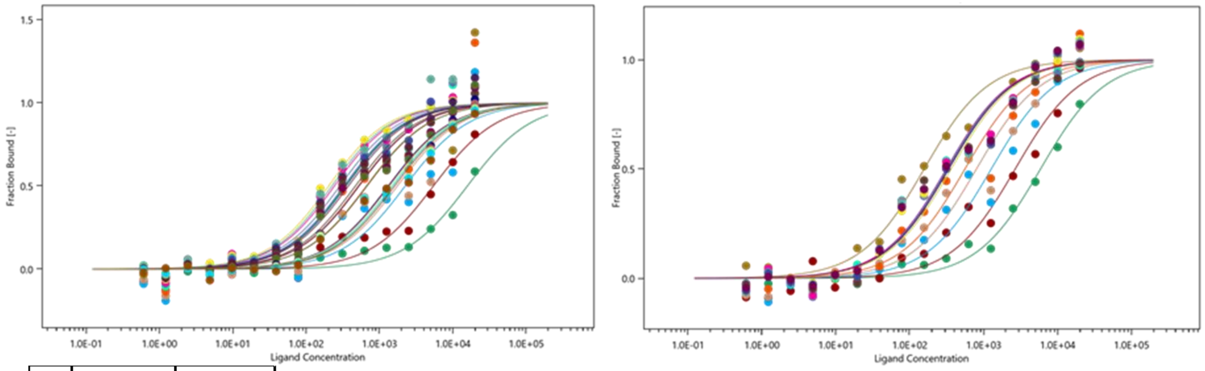

t (h)	$pK_{D(MUC4)}$	$pK_{D(EGF312)}$
0	4,20	3,77
0,5	3,73	3,43
1	3,35	3,10
1,5	3,24	2,90
2	2,81	2,74
2,5	2,69	
3	2,50	
3,5	2,42	
4	2,34	
4,5	2,25	2,13
5	2,30	2,12
5,5	2,48	2,14
6	2,51	2,29
6,5	2,55	2,27
7	2,65	2,43
7,5	2,70	2,50
8	2,81	2,51
8,5	3,11	2,61
9	3,12	
9,5	3,15	
10	3,17	
10,5	3,20	3,00

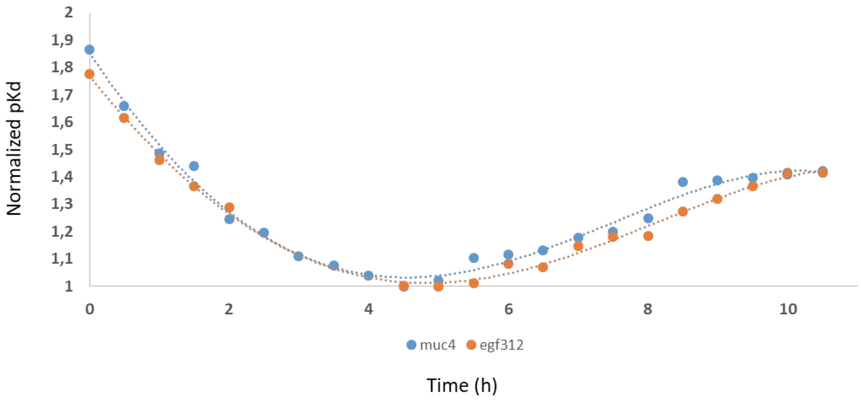

Impact of the incubation time on the MUC4β-ErbB2 K_d measurement. Evaluation of the interaction kinetics for MUC4β (top left) and $MUC4_{EGF3+1+2}$ (top right) proteins with ErbB2, at 80% MST power. Affinity is monitored every 30 min at room temperature. pK_d Data, normalized by the lower value, are plotted below and show a kinetic for the interaction with an optimal K_d found after at least 3 hours of incubation . After 7h, degradation of the lysate is observed at room temperature. The same kinetic is found with both proteins.

SPR studies

eGFP capture. To develop a label-free strategy of SPR, we used the eGFP-MUC4 fusion proteins to ensure specific capture from the lysate. CHO-K1 cell lysates expressing either eGFP-MUC4β or eGFP-MUC4$_{EGF3+1+2}$ were thus prepared in MPer buffer, as for the MST studies. A step-by-step development was chosen to quickly identify any problems and therefore eGFP was first used. Two strategies of capture (direct or indirect) of pure eGFP using specific antibodies were assayed. Unfortunately, none of these methods led to a response signal following eGFP injection (not shown). As these antibodies are often used for biomolecular assays with longer incubation time, they appeared not suited for SPR assays.

Recently, a single domain antibody was described as a potent specific and stable GFP-binding protein (GBP) usable for efficient fishing of GFP tagged proteins. Moreover, the use of GBP from complex biological samples such as cell extracts[1] is very convenient in a Biacore® capture assay format. The immobilization of GBP on the sensor chip following the procedure described by Biacore® was realized to produce a GBP coated chip (CM5 sensor chip with covalently bound GBP).[2] First, 3458 RU of GBP were chip-immobilized, and then, another chip was produced with 554 RU (a lower level as advised for binding experiments between two proteins). Solutions of pure eGFP with concentrations ranging from 2 pM to 200 nM were injected and the GBP coated chip was able to detect eGFP from 2 pM and the saturation reached 85% of the theoretical maximum response at 200 nM (Fig.S4A). Regeneration was efficiently done by injection of Glycine-HCl buffer (0.01 M, pH 2.0) and this step allowed highly reproducible measurements with 0.25% of variability coefficient (VC) (Fig.S4B). Diluted pure eGFP in CHO-K1 cell extract was also injected to evaluate the impact of a crude lysate on the binding property of the GBP chip and no major perturbation was observed (Fig.S4A, light blue curve). Altogether, these data validated our method.

Capture of eGFP-MUC4β. eGFP-MUC4β expressing CHO-K1 cells were lysed in MPer buffer as previously described. Cell lysates were then diluted once to obtain a constant concentration of 50 nM of eGFP-MUC4β (measurement of the intensity of fluorescence of eGFP reported on a titration curve as described in the material & methods section) and injected on the Biacore® system. Capture of the proteins was monitored with an increase of the SPR signal during association phase followed by efficient regeneration steps with a VC of 0.72% (Fig.S4C).

MUC4β-ErbB2-Fc interaction study. On the GBP-coated sensor chip with immobilized eGFP-MUC4β, we then investigated the binding of ErbB2 with increasing recombinant ErbB2-Fc concentrations (2, 20, 200 nM and up to 2µM). This did not lead to any results (Fig.S5). As the MST studies were successfully carried out with a pre-incubation time, we decided to design SPR assays with a preliminary incubation of 200 nM ErbB2 with CHO-K1 cell lysates expressing eGFP-MUC4β. A significant enhancement ($p<0.005$ on $n=5$) of the signal indicated the binding of a protein with a higher molecular weight than eGFP-MUC4β. This result suggested that the eGFP-MUC4β/ErbB2 complex formation and immobilization on the GBP-coated sensor chip occurred (Fig.S6). Moreover, this response was specific as a pre-incubation with non-relevant Fc-protein (PD-1-Fc) did not lead to any significant change of the response.

Discussion

Our strategy merges SPR sensitivity to detect proteins from biological fluids with the single domain antibody GBP as a powerful tool to immobilize eGFP-MUC4β fusion protein from cell lysate.[1,2] Very good results were obtained regarding the immobilization of MUC4β from the lysates followed by an easy and reproducible regeneration step. As binding studies with increasing recombinant ErbB2-Fc concentrations were performed without success, we

attempted to directly fish the MUC4β-ErbB2 complex after a pre-incubation step. We considered that the level of the residual signal after injection of the cell lysate followed by washing procedure with running buffer to remove all non-specific interacting proteins, could approximately estimate the amount of the fished complex that can be caught from the lysate. Our data were in accordance with recent works in SPR with cell lysates[3], describing sensorgrams with similar profiles. The maximum binding responses (Rmax) observed were low compared to the expected values according to the high molecular weight of the complex. However, these results suggested the specific eGFP-MUC4β/ErbB2 complex formation and immobilization on the GBP-coated sensor chip (Fig. S6). MUC4β-ErbB2 dose-ranging studies were performed with pre-incubation of various concentrations of recombinant ErbB2-Fc and constant concentrations of eGFP-MUC4β cell lysate. Low binding responses were observed during association phases but without the opportunity to quantitatively estimate the binding affinity. Compared to MST, SPR needs high ErbB2 sample consumption and does not appear to be adapted for studying the specific characteristics of the MUC4β-ErbB2 which needs long incubation times not appropriate for a microfluidic system.

Methods

Surface Plasmon Resonance – Surface preparation. Surface Plasmon Resonance (SPR) assay was carried out following the GFP-Trap® (gt-250 GFP-Binding protein, Chromotek, Germany) capture surface for Biacore assays protocol. SPR measurement was performed using a BIAcore 3000 instrument with four flow channels. GFP Binding Protein (GBP) was immobilized covalently on a CM5 sensor chip by amine coupling according to the method described by Wu *et al.*[4] The affinity of GBP for GFP is extremely high, with an equilibrium dissociation constant of about 10^{-12} M. Hence, the eGFP-tagged protein can be captured by the GBP on the surface of the sensor chip. The surface of the CM5 sensor chip is covered with a

matrix of carboxymethylated dextran, a flexible unbranched carbohydrate polymer forming a thin surface layer. For the immobilization of GBP, 10 mM acetate buffer (pH 5.5) was used as a dilution buffer, and 1X PBS with 0.005 % (v/v) P20 buffer was used as a running buffer at a flow rate of 10 μL.min^{-1}. The surface was activated by injecting the mixture containing equal volumes of 0.1 M N-hydroxysuccinimide (NHS) and 0.4 M 1-ethyl-3-(3-dimethylaminopropyl) carbodiimide (EDC) to generate reactive succinimide esters for 7 min. 50 μg.mL^{-1}. GBP was passed over the surface and the primary amine groups of GBP reacted spontaneously with the succinimide esters on the surface of the sensor chip. The excess reactive groups were blocked by injecting 1 M ethanolamine-HCl (pH 8.5) at a flow rate of 10 μL.min^{-1} for 7 min. The online response was applied to characterize the sensor surface. Final immobilization levels were expressed as resonance units (RU). Various concentrations of eGFP and eGFP-tagged proteins were injected and captured onto the surface of channel 2 only. The reference channel 1 without GBP was prepared following the same immobilization procedure.

SPR measurement. The instrument system was equilibrated with the running buffer (pH = 7.4) at 25 ± 0.1 °C. Different concentrations of purified eGFP (2 pM – 200 nM) were prepared by serial dilutions in running buffer. To evaluate and compare the impact of crude lysate, purified eGFP (200 nM) was also diluted in empty lysate. Crude lysate with eGFP tagged proteins were at least diluted in an equal volume of running buffer to a minimal concentration of eGFP-MUC4β of 50 nM. ErbB2 solutions were prepared by serial dilutions and injected from the lower to the higher concentration of 2 µM for direct titration at 10µL/min for 8 min and 2 min between each injection. For the specificity of the interaction, solutions of 100 nM eGFP-tagged lysate and 400 nM ErbB2-Fc or PD1-Fc were mixed at equal volume and incubated 3h before injection. For the titration attempt with pre-incubation, solutions of ErbB2 were mixed with eGFP-tagged MUC4β lysates. The final concentration in eGFP-MUC4β was kept to 50 nM and the final concentration of ErbB2 ranged from 4.02 µM to 53 nM following a ratio of 3:1 dilution

in the lysate. After incubation, each solution was injected in the instrument. The data was analyzed using the BIAcore Evaluation Software (GE Healthcare, USA).

References

1. Kubala, M. H., Kovtun, O., Alexandrov, K. & Collins, B. M., Structural and thermodynamic analysis of the GFP:GFP-nanobody complex. *Protein Sci.* **12** (19), 2389-401 (2010).

2. Chromotek, GFP-Trap Capture surface, Available at HYPERLINK "https://www.chromotek.com/fileadmin/user_upload/pdfs/Application_notes/LabGuide-GFPTrapCaptureSurface.pdf"

3. Florinskaya, A. et al., SPR Biosensors in Direct Molecular Fishing: Implications for Protein Interactomics. *Sensors* (Basel) **18** (5), E1616 (2018).

4. Wu, W. et al., A direct determination of AFBs in vinegar by aptamer-based surface plasmon resonance biosensor. *Toxicon*. **146**, 24-30 (2018).

Supplementary Fig. S4

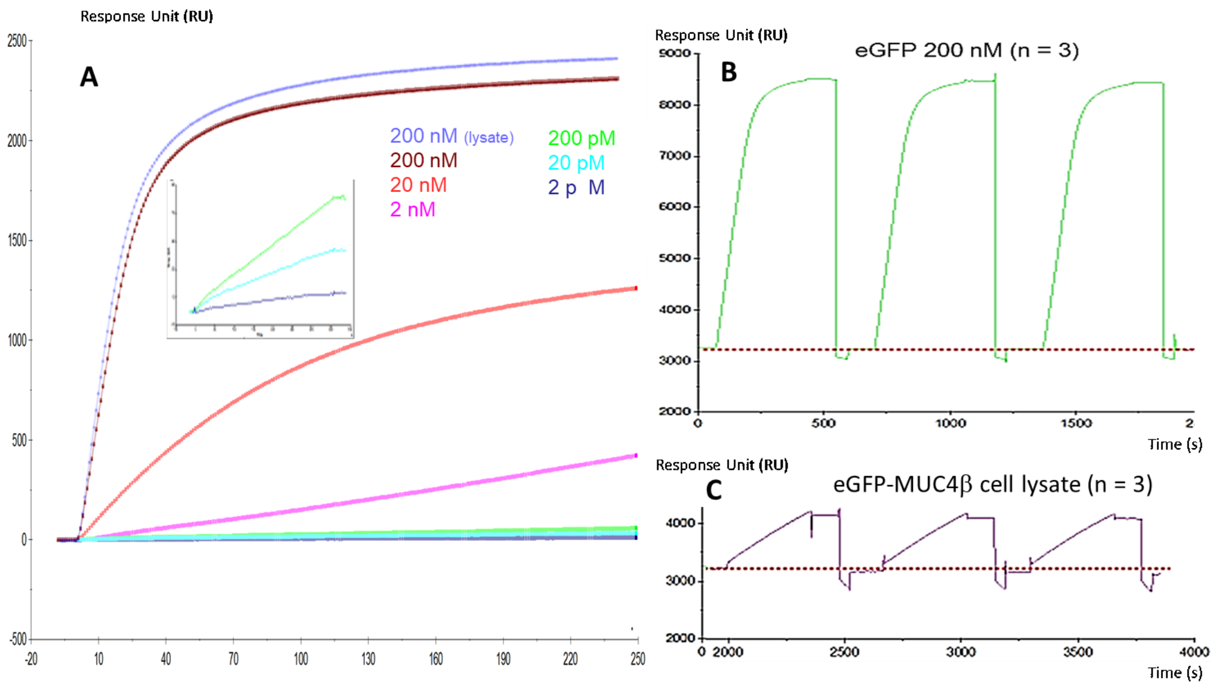

Capture efficiency of the GBP coated chip by SPR. (A) Binding curves of pure eGFP to immobilized GBP from 2 pM to 200 nM in PBS, with a zoom for the picomolar binding curves. The saturation for 200 nM of eGFP reaches 85% of the theoretical maximum occupancy. In light blue, the curve for 200 nM of eGFP diluted in cell lysate shows similar profile and saturation. (B) Triplicate performed at 200 nM of eGFP and with a regeneration step shows high reproducibility with a VC of 0.25%. (C) Triplicate performed on a full cell lysate of eGFP-MUC4β and with a regeneration step shows again a high reproducibility with a VC of 0.72%.

Supplementary Fig. S5

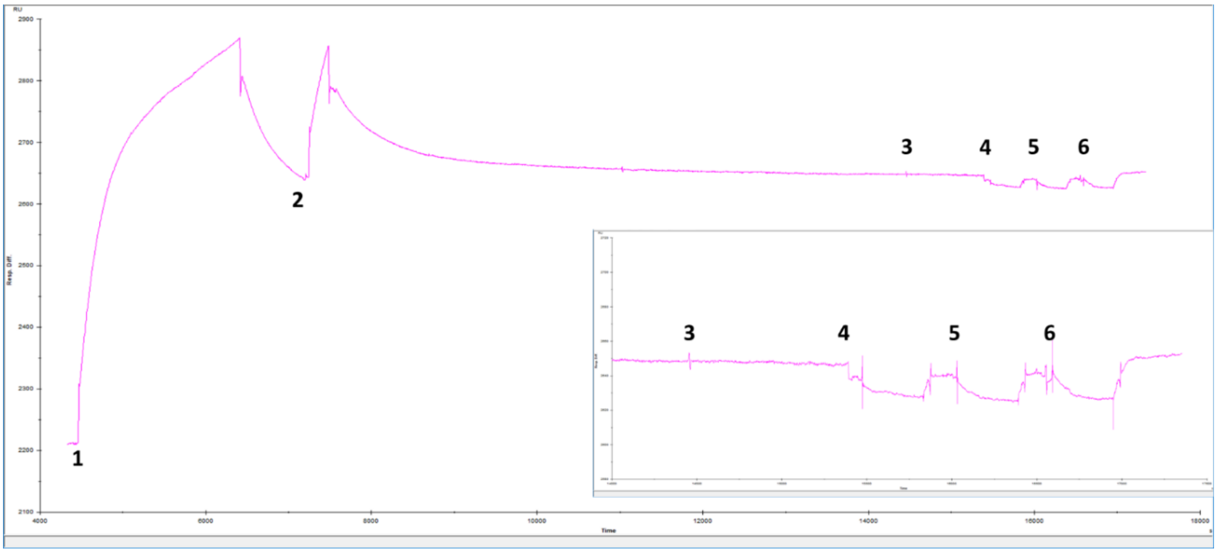

Injection of increasing concentrations of recombinant ErbB2 on eGFP-MUC4β-GBP immobilized chip. In 1 and 2, lysate containing eGFP-MUC4β was injected to be immobilized on the GBP-coated chip. After the second injection, buffer was run to ensure the stability of the immobilized MUC4β. Then in 3, 4, 5 and 6 was injected increasing concentration of recombinant ErbB2-Fc in PBS (respectively 2, 20, 200 nM and 2 µM). No binding could be observed in these conditions.

Supplementary Fig. S6

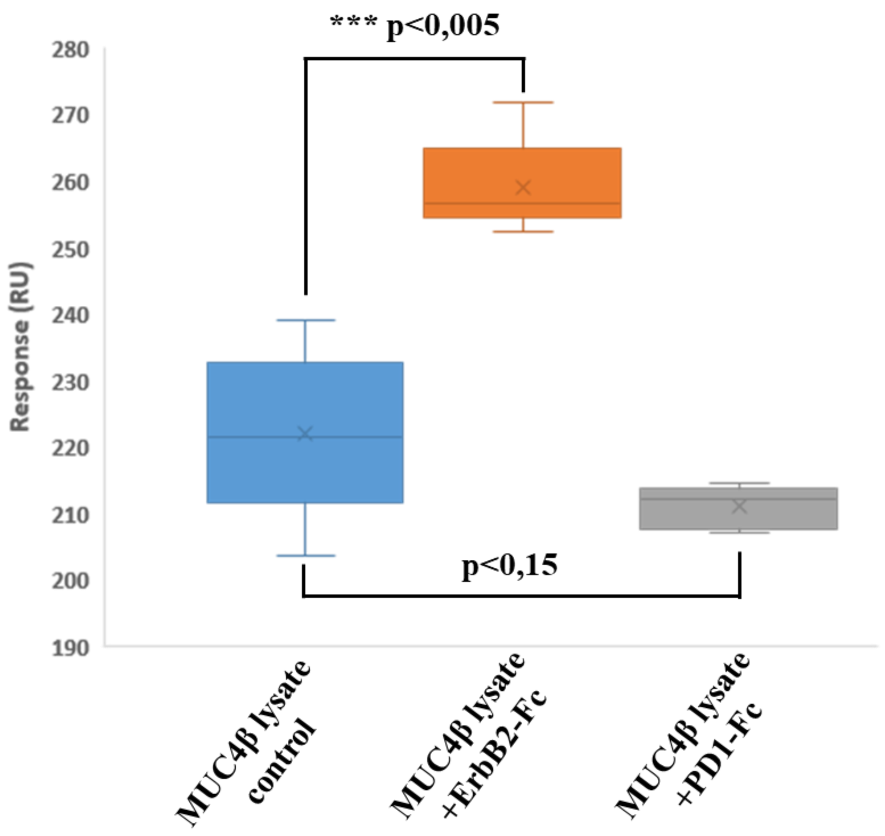

Specific detection of the interaction between eGFP-MUC4β and ErbB2-Fc after incubation by SPR. Lysates were incubated 3h at room temperature , either alone (blue), with 200 nM of ErbB2-Fc (orange) or with non-relevant equivalent PD-1-Fc protein (grey) then injected on GBP immobilized chip. The mean value for the RU response between 440 and 500 s after injection was plotted in n=5 independent injections in a box plot representation. The lysate containing MUC4 presents a significant increase (*** p<0.005) of the response with incubation of ErbB2-Fc in comparison to the same lysate with no protein incubated or with incubation of a negative control, PD1-Fc (p<0.0001). A difference was observed between the negative control and the sole lysate but was not significate (p<0.12).

Supplementary Fig. S7

```
TAGTTATTAATAGTAATCAATTACGGGGTCATTAGTTCATAGCCCATATATGGAGTTCCGCGTTACATAACTTACGGTAAATGGCCCGCCTGGCTGACCGCCCAACGACCCCCGCCCATTGACGTCAATAATGACGTATGTTCCCATAGTAACGCCAATAGGGACTTTCCATTGACG
TCAATGGGTGGAGTATTTACGGTAAACTGCCCACTTGGCAGTACATCAAGTGTATCATATGCCAAGTACGCCCCCTATTGACGTCAATGACGGTAAATGGCCCGCCTGGCATTATGCCCAGTACATGACCTTATGGGACTTTCCTACTTGGCAGTACATCTACGTATTAGTCATCGC
TATTACCATGGTGATGCGGTTTTGGCAGTACATCAATGGGCGTGGATAGCGGTTTGACTCACGGGGATTTCCAAGTCTCCACCCCATTGACGTCAATGGGAGTTTGTTTTGGCACCAAAATCAACGGGACTTTCCAAAATGTCGTAACAACTCCGCCCCATTGACGCAAATGG
GCGGTAGGCGTGTACGGTGGGAGGTCTATATAAGCAGAGCTGGTTTAGTGAACCGTCAGATCCGCTAGCGCTACCGGTCGCCACCATGGTGAGCAAGGGCGAGGAGCTGTTCACCGGGGTGGTGCCCATCCTGGTCGAGCTGGACGGCGACGTAAACGGCCACAAGTTC
AGCGTGTCCGGCGAGGGCGAGGGCGATGCCACCTACGGCAAGCTGACCCTGAAGTTCATCTGCACCACCGGCAAGCTGCCCGTGCCCTGGCCCACCCTCGTGACCACCCTGACCTACGGCGTGCAGTGCTTCAGCCGCTACCCCGACCACATGAAGCAGCACGACTTCTT
CAAGTCCGCCATGCCCGAAGGCTACGTCCAGGAGCGCACCATCTTCTTCAAGGACGACGGCAACTACAAGACCCGCGCCGAGGTGAAGTTCGAGGGCGACACCCTGGTGAACCGCATCGAGCTGAAGGGCATCGACTTCAAGGAGGACGGCAACATCCTGGGGCACAA
GCTGGAGTACAACTACAACAGCCACAACGTCTATATCATGGCCGACAAGCAGAAGAACGGCATCAAGGTGAACTTCAAGATCCGCCACAACATCGAGGACGGCAGCGTGCAGCTCGCCGACCACTACCAGCAGAACACCCCCATCGGCGACGGCCCCGTGCTGCTGCCCG
ACAACCACTACCTGAGCACCCAGTCCGCCCTGAGCAAAGACCCCAACGAGAAGCGCGATCACATGGTCCTGCTGGAGTTCGTGACCGCCGCCGGGATCACTCTCGGCATGGACGAGCTGTACAAGTCCGGACTCAGATCTATGAAGGGCGCCAGATGGCGGAGAGTGCCT
TGGGTTTCACTGTCCTGCCTGTGCCTGTGTCTGCTGCCTCACGTGGTGCCCAATCCTCACATCACCACACTGGACGGCGTGTCCTACACCTTCAATGGCCTGGGCGATTTCCTGCTCGTGGGCGCTCAGGATGGCAACTCTAGCTTTCTGCTGCAAGGCCGGACAGCCCAGAC
AGGATCTGCTCAGGCCACCAACTTTATCGCCTTCGCCGCTCAGTACCGGTCCTCTTCTCTGGGACCTGTGACAGTGCAGTGGCTGCTGGAACCTCACGACGCCATTAGAGTGCTGCTGGACAACCAGACCGTGACCTTCCAGCCTGATCACGAAGATGGCGGCGGACAAGA
GACATTCAATGCCACAGGCGTGCTGCTGTCCCGGAATGGATCTGAGGTGTCCGCCTCTTTTGATGGCTGGGCCACCGTGTCTGTGATCGCCCTGTCTAATATCCTGCACGCCTCTGCCAGTCTGCCTCCTGAGTACCAGAATAGAACCGAGGGCCTGCTCGGCGTGTGGAACA
ACAACCCTGAGGACGACTTCCGGATGCCTAACGGCTCTACAATCCCTCCTGGCAGCCCCGAGGAAATGCTGTTCCACTTTGGCATGACCTGGCAGATCAACGGCACCGGACTGCTGGGCAAGAGAAACGACCAGCTGCCTTCCAACTTCACCCCTGTGTTCTACAGCCAGCT
GCAGAAGAACTCCTCCTGGGCCGAGCACCTGATCTCTAACTGCGACGGCGACTCCAGCTGCATCTACGATACCCTGGCTCTGCGGAACGCCTCTATCGGCCTGCATACAAGAGAGGTGTCCAAGAACTACGAGCAGGCTAACGCTACCCTGAATCAGTACCCTCCTTCCATCA
ACGGCGGCAGAGTGATCGAGGCTTACAAGGGCCAGACCACACTGATCCAGTACACCTCCAATGCCGAGGACGCCAACTTTACCCTGCGGGACTCTTGCACCGACCTGGAACTGTTCGAGAACGGCACCCTGCTGTGGACCCCTAAGAGCCTGGAACCTTTCACACTGGAA
ATCCTGGCCAGATCCGCCAAGATCGGCCTGGCTTCTGCTCTGCAGCCTAGAACCGTCGTGTGCCACTGTAACGCCGAGAGCCAGTGCCTGTACAATCAGACCTCCAGAGTGGGCAACTCCAGCCTGGAAGTGGCTGGCTGCAAATGTGATGGCGGCACCTTCGGCAGATAC
TGCGAGGGCTCTGAAGATGCCTGCGAGGAACCTTGCTTCCCCTCTGTGCATTGTGTGCCTGGCAAGGGCTGTGAAGCCTGTCCTCCTAATCTGACCGGCGACGGCAGACACTGTGCTGCTCTGGGATCTAGCTTCCTGTGCCAGAACCAGAGCTGCCCCGTGAACTACTGCT
ACAACCAGGGCCACTGCTACATCTCTCAGACCCTGGGATGCCAGCCTATGTGCACCTGTCCACCAGCCTTTACCGACAGCAGATGTTTCCTGGCCGGCAACAACTTCTCCCCTACCGTGAATCTGGAACTGCCCCTGAGAGTGATCCAGCTGCTGCTGAGCGAGGAAGAGAA
TGCCTCCATGGCCGAAGTGAACGCCTCCGTGGCTTATAGACTGGGCACCCTGGATATGCGGGCCTTCCTGAGAAACTCCCAGGTGGAACGGATCGACTCTGCTGCTCCTGCTTCCGGCTCTCCTATCCAGCACTGGATGGTCATCTCCGAGTTCCAGTACCGGCCTAGAGGCC
CCGTGATCGACTTCCTGAACAATCAGCTGCTGGCCGCCGTGGTGGAAGCCTTCCTGTATCACGTGCCCAGAAGATCCGAGGAACCCCGGAACGACGTGGTGTTCCAGCCTATCTCTGGCGAGGATGTGCGGGATGTGACCGCTCTGAATGTGTCCACACTGAAGGCCTACTT
CAGATGCGACGGCTACAAGGGCTACGACCTGGTGTACTCTCCCCAGTCCGGCTTCACCTGTGTGTCTCCCTGTTCCAGAGGCTACTGTGATCATGGCGGCCAGTGCCAGCATCTGCCTTCCGGACCTAGATGCTCCTGCGTGTCCTTCTCCATCTACACCGCTTGGGGCGAGC
ACTGCGAGCATCTGTCTATGAAGCTGGACGCCTTCTTCGGCATCTTCTTTGGCGCTCTCGGAGGCCTGCTGCTGCTTGGAGTGGGAACATTCGTGGTGCTGAGATTCTGGGGCTGTAGCGGCGCCAGGTTCTCCTACTTTCTGAACTCTGCCGAGGCTCTGCCCGGATCCACC
GGATCTAGATAACTGATCATAATCAGCCATACCACATTTGTAGAGGTTTTACTTGCTTTAAAAAACCTCCCACACCTCCCCCTGAACCTGAAACATAAAATGAATGCAATTGTTGTTGTTAACTTGTTTATTGCAGCTTATAATGGTTACAAATAAAGCAATAGCATCACAAATTTCA
CAAATAAAGCATTTTTTTCACTGCATTCTAGTTGTGGTTTGTCCAAACTCATCAATGTATCTTAACGCGTAAATTGTAAGCGTTAATATTTTGTTAAAATTCGCGTTAAATTTTTGTTAAATCAGCTCATTTTTTAACCAATAGGCCGAAATCGGCAAAATCCCTTATAAATCAAAAGA
ATAGACCGAGATAGGGTTGAGTGTTGTTCCAGTTTGGAACAAGAGTCCACTATTAAAGAACGTGGACTCCAACGTCAAAGGGCGAAAAACCGTCTATCAGGGCGATGGCCCACTACGTGAACCATCACCCTAATCAAGTTTTTTGGGGTCGAGGTGCCGTAAAGCACTAAAT
CGGAACCCTAAAGGGAGCCCCCGATTTAGAGCTTGACGGGGAAAGCCGGCGAACGTGGCGAGAAAGGAAGGGAAGAAAGCGAAAGGAGCGGGCGCTAGGGCGCTGGCAAGTGTAGCGGTCACGCTGCGCGTAACCACCACACCCGCCGCGCTTAATGCGCCGCTACA
GGGCGCGTCAGGTGGCACTTTTCGGGGAAATGTGCGCGGAACCCCTATTTGTTTATTTTTCTAAATACATTCAAATATGTATCCGCTCATGAGACAATAACCCTGATAAATGCTTCAATAATATTGAAAAAGGAAGAGTCCTGAGGCGGAAAGAACCAGCTGTGGAATGTGTGT
CAGTTAGGGTGTGGAAAGTCCCCAGGCTCCCCAGCAGGCAGAAGTATGCAAAGCATGCATCTCAATTAGTCAGCAACCAGGTGTGGAAAGTCCCCAGGCTCCCCAGCAGGCAGAAGTATGCAAAGCATGCATCTCAATTAGTCAGCAACCATAGTCCCGCCCCTAACTCCGC
CCATCCCGCCCCTAACTCCGCCCAGTTCCGCCCATTCTCCGCCCCATGGCTGACTAATTTTTTTTATTTATGCAGAGGCCGAGGCCGCCTCGGCCTCTGAGCTATTCCAGAAGTAGTGAGGAGGCTTTTTTGGAGGCCTAGGCTTTTGCAAAGATCGATCAAGAGACAGGATG
AGGATCGTTTCGCATGATTGAACAAGATGGATTGCACGCAGGTTCTCCGGCCGCTTGGGTGGAGAGGCTATTCGGCTATGACTGGGCACAACAGACAATCGGCTGCTCTGATGCCGCCGTGTTCCGGCTGTCAGCGCAGGGGCGCCCGGTTCTTTTTGTCAAGACCGACCT
GTCCGGTGCCCTGAATGAACTGCAAGACGAGGCAGCGCGGCTATCGTGGCTGGCCACGACGGGCGTTCCTTGCGCAGCTGTGCTCGACGTTGTCACTGAAGCGGGAAGGGACTGGCTGCTATTGGGCGAAGTGCCGGGGCAGGATCTCCTGTCATCTCACCTTGCTCCTG
CCGAGAAAGTATCCATCATGGCTGATGCAATGCGGCGGCTGCATACGCTTGATCCGGCTACCTGCCCATTCGACCACCAAGCGAAACATCGCATCGAGCGAGCACGTACTCGGATGGAAGCCGGTCTTGTCGATCAGGATGATCTGGACGAAGAGCATCAGGGGCTCGCGCC
AGCCGAACTGTTCGCCAGGCTCAAGGCGAGCATGCCCGACGGCGAGGATCTCGTCGTGACCCATGGCGATGCCTGCTTGCCGAATATCATGGTGGAAAATGGCCGCTTTTCTGGATTCATCGACTGTGGCCGGCTGGGTGTGGCGGACCGCTATCAGGACATAGCGTTGGC
TACCCGTGATATTGCTGAAGAGCTTGGCGGCGAATGGGCTGACCGCTTCCTCGTGCTTTACGGTATCGCCGCTCCCGATTCGCAGCGCATCGCCTTCTATCGCCTTCTTGACGAGTTCTTCTGAGCGGGACTCTGGGGTTCGAAATGACCGACCAAGCGACGCCCAACCTGCC
ATCACGAGATTTCGATTCCACCGCCGCCTTCTATGAAAGGTTGGGCTTCGGAATCGTTTTCCGGGACGCCGGCTGGATGATCCTCCAGCGCGGGGATCTCATGCTGGAGTTCTTCGCCCACCCTAGGGGGAGGCTAACTGAAACACGGAAGGAGACAATACCGGAAGGAAC
CCGCGCTATGACGGCAATAAAAAGACAGAATAAAACGCACGGTGTTGGGTCGTTTGTTCATAAACGCGGGGTTCGGTCCCAGGGCTGGCACTCTGTCGATACCCCACCGAGACCCCATTGGGGCCAATACGCCCGCGTTTCTTCCTTTTCCCCACCCCACCCCCCAAGTTCG
GGTGAAGGCCCAGGGCTCGCAGCCAACGTCGGGGCGGCAGGCCCTGCCATAGCCTCAGGTTACTCATATATACTTTAGATTGATTTAAAACTTCATTTTTAATTTAAAAGGATCTAGGTGAAGATCCTTTTTGATAATCTCATGACCAAAATCCCTTAACGTGAGTTTTCGTTCC
ACTGAGCGTCAGACCCCGTAGAAAAGATCAAAGGATCTTCTTGAGATCCTTTTTTTCTGCGCGTAATCTGCTGCTTGCAAACAAAAAAACCACCGCTACCAGCGGTGGTTTGTTTGCCGGATCAAGAGCTACCAACTCTTTTTCCGAAGGTAACTGGCTTCAGCAGAGCGCA
GATACCAAATACTGTCCTTCTAGTGTAGCCGTAGTTAGGCCACCACTTCAAGAACTCTGTAGCACCGCCTACATACCTCGCTCTGCTAATCCTGTTACCAGTGGCTGCTGCCAGTGGCGATAAGTCGTGTCTTACCGGGTTGGACTCAAGACGATAGTTACCGGATAAGGCGCA
GCGGTCGGGCTGAACGGGGGGTTCGTGCACACAGCCCAGCTTGGAGCGAACGACCTACACCGAACTGAGATACCTACAGCGTGAGCTATGAGAAAGCGCCACGCTTCCCGAAGGGAGAAAGGCGGACAGGTATCCGGTAAGCGGCAGGGTCGGAACAGGAGAGCGCA
CGAGGGAGCTTCCAGGGGGAAACGCCTGGTATCTTTATAGTCCTGTCGGGTTTCGCCACCTCTGACTTGAGCGTCGATTTTTGTGATGCTCGTCAGGGGGGCGGAGCCTATGGAAAAACGCCAGCAACGCGGCCTTTTTACGGTTCCTGGCCTTTTGCTGGCCTTTTGCTCA
CATGTTCTTTCCTGCGTTATCCCCTGATTCTGTGGATAACCGTATTACCGCCATGCAT
```

Full length vector of MUC4β. Full length vector of the peGFP-C1 kana/neo MUC4β, with the cloning sites BglII and BamHI used and a codon optimization for CHO cells performed.
